# Deciphering the vectors: Unveiling the local dispersal of *Litylenchus crenatae* ssp. *mccannii* in the American beech (*Fagus grandifolia*) forest ecosystem

**DOI:** 10.1371/journal.pone.0311830

**Published:** 2024-11-08

**Authors:** Mankanwal Goraya, Camelia Kantor, Paulo Vieira, Danielle Martin, Mihail Kantor

**Affiliations:** 1 Plant Pathology and Environmental Microbiology Department, The Pennsylvania State University, University Park, Pennsylvania, United States of America; 2 Huck Institutes of the Life Sciences, The Pennsylvania State University, University Park, Pennsylvania, United States of America; 3 Mycology and Nematology Genetic Diversity and Biology Laboratory, United States Department of Agriculture, Agricultural Research Service, Beltsville, Maryland, United States of America; 4 United States Department of Agriculture, Forest Service, Forest Health Protection, Morgantown, West Virginia, United States of America; University of Limpopo, SOUTH AFRICA

## Abstract

Beech leaf disease (BLD), caused by the *Litylenchus crenatae* ssp. *mccannii* (Lcm) nematode, is an emerging threat to beech trees. This disease is characterized by distinct leaf symptoms, including leaf interveinal banding and thickened leaf texture, which leads to eventual tree mortality. Understanding Lcm dispersal mechanism(s) is crucial for managing BLD, yet these remain largely unknown, posing a major barrier to its effective management. This study represents a pioneering investigation into the abiotic and biotic vectors that potentially contribute to the local dispersal of Lcm in natural American beech (*Fagus grandifolia*) forest systems in the Northeastern United States. An experiment was set up in Stone Valley Forest, Pennsylvania (PA), using four funnel stands placed at variable distances from naturally BLD-infected beech trees. This approach enabled the recovery of active Lcm nematodes from each funnel, demonstrating their ability to naturally disperse at least 11.74 m from the nearest BLD-infected tree. The findings highlight the role of abiotic factors involved in the dispersal dynamics of Lcm, especially wind and humidity, as indicated by a generalized linear model. The current study also uncovered the incidental association of Lcm with other organisms beneath the canopy of BLD trees, including spiderwebs and caterpillars. To our knowledge, this is the first study to document the potential vectors involved in the local dispersal of Lcm, offering valuable information for the biology of this nematode, as well as insight into the development of effective BLD management strategies. The findings contribute to broader efforts in advancing the understanding of the local spread of BLD, highlighting the complex interplay of abiotic and biotic factors in this disease dispersal.

## Introduction

Beech trees (*Fagus* spp.) are important species with ecological significance worldwide. *Fagus grandifolia* (Ehrh.) is considered one of the dominant trees of the Northeastern hardwood forests in North America [1]. The European beech, *Fagus sylvatica* L., is known for its vital role in nutrient cycling, carbon storage, and controlling erosion [2–4]. Many wild animals, such as squirrels, wild turkeys, and white-tailed deer have been reported to be dependent on beechnuts for fats and proteins [5]. Moreover, some insects and birds rely on beech tree canopies for shelter and nesting [5].

Throughout North America, the American beech, is facing a major threat from the lethal beech leaf disease (BLD). The causal agent of BLD is *Litylenchus crenatae* ssp. *mccannii* (Lcm), a recently described foliar nematode, affecting the foliage and buds of beech trees [6]. BLD was first reported at Lake Metroparks, Lake County, Ohio, in the United States in 2012 [7]. In addition to *F*. *grandifolia*, *F*. *sylvatica*, Chinese beech (*F*. *engleriana* Seemen ex Diels), and Oriental beech (*F*. *orientalis* Lipsky) have also been reported to be susceptible to BLD [8]. Subsequently, BLD has rapidly spread in beech trees of the Northeastern United States and in Ontario, Canada, presenting a considerable threat to *Fagus* spp. [7, 9–13]. Since BLD poses a significant risk to beech trees, it is important to understand the vectors involved in Lcm’s dispersal dynamics.

*Litylenchus crenatae* ssp. *mccannii* is a member of the Anguinidae family, which includes mycophagous and plant-parasitic nematodes (PPNs) [13, 15]. Within this family, several species are considered significant pests and classified as quarantine nematodes in various areas of the world, e.g., *Ditylenchus* and *Anguina* species, due to their negative effects in agriculture [14]. These nematodes can infect aerial parts of the plants, causing swellings and galls [14, 15]. Their host preferences vary, with some species exhibiting a broad range of hosts, while others displaying narrow host specificity [14]. As migratory nematodes, they can move across the surface of host tissues using water films to migrate, frequently parasitizing leaves, stems, inflorescences and seeds, and occasionally roots [14, 16]. Typically, the infective-stage juveniles penetrate the host tissues (e.g., buds, lenticels, stomata, petioles) and then migrate intercellularly [15]. Once established in the plant tissues, members of this family can induce host cell hyperplasia and hypertrophy, resulting in leaf or bulb deformities, shorter internodes, and neoplastic tissues [14, 15].

The life cycle of Lcm is highly synchronized with the development of the bud and inner developing leaves [14]. As temperatures drop and leaves senesce in the fall, Lcms migrate from leaves to buds [14]. Once established within the bud tissues, nematodes use bud scales and newly forming leaves as sources of nutrients to develop and increase their population numbers [14]. As buds enter dormancy to survive the winter, Lcms use the bud as a closed structure to shield themselves from adverse environmental conditions [14]. Members of this family are also known for their specialized cryptobiotic state which allows them to survive adverse abiotic conditions [17]. For example, *Ditylenchus dipsaci*, often survives on seeds and dry plant debris within heavily infested plants and plays a very important role in the passive dissemination of the nematode over long distances [17].

The natural dissemination of nematodes often involves specific associations with biological vector(s) (e.g., insects), and can be influenced directly by environmental conditions. For instance, heavy rainfall can play a significant role in the dissemination of PPNs through runoff and flooding [18]. In addition, wind is also reported to act as a natural dispersal agent for cyst nematodes (*Heterodera* spp. and *Globodera* spp.) [16–18]. Apart from indirect methods of dispersion, certain nematodes are also highly mobile. For example, foliar nematodes (*Aphelenchoides ritzemabosi*, Family Aphelenchoididae), can actively move at speeds up to 1.5 cm/minute [19]. The distance they can reach can also be increased by their dispersal through water splashes due to overhead watering systems, water dripping from taller plants, or by moisture films generated by dew [20]. Within the family Anguinidae, different spread mechanisms have been reported, such as trade of infected seeds and crop straw, flooding, rainfall and wind [17]. Also, in its desiccated state on infected seeds, some species of this family (e.g., *Ditylenchus dipsaci*) can survive passage through animals’ gastrointestinal digestive tract (e.g., domestic livestock, insects, and birds) [17, 18, 21]. In the case of Lcm, nematodes have been previously detected on the leaf surface and twigs of BLD symptomatic trees, highlighting their ability to move effectively [18]. Furthermore, Lcm can reach high densities (thousands of nematodes per leaf) by late summer and early fall [14], increasing the likelihood of exposure to environmental conditions.

Until recently, there has been limited evidence concerning the dispersal mechanisms of Lcm. This prompted us to investigate the timing of peak migration of Lcm nematodes and how biotic and abiotic factors influence their local-scale dispersal. Therefore, the primary aim of this study was to elucidate the potential vectors responsible for the local dispersal of Lcm. In pursuit of this goal, we assessed how abiotic environmental conditions can impact Lcm dispersal and explored their incidental association with various organisms commonly found in the American beech forest ecosystems.

## Material and methods

### Experimental set-up

**Fig 1:**
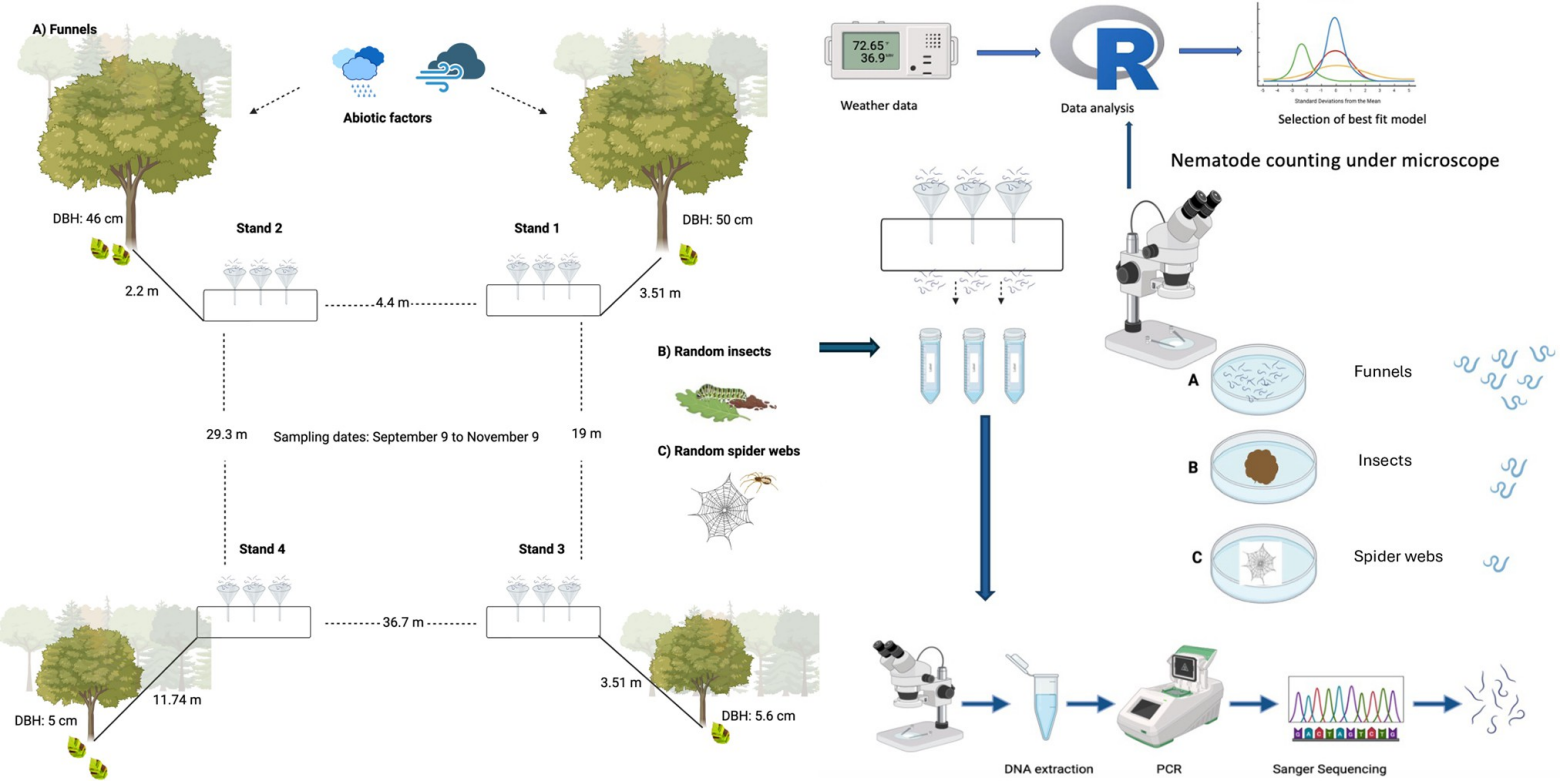
Schematic illustration of the experimental sampling procedure implemented in this study. Four funnel stands were rigorously set up at varying distances from BLD symptomatic trees. Each funnel stand consisted of three water-filled funnels. Nematodes were then recovered from funnels (A), random insects (B), and random spider webs (C) located under the canopy of BLD-infected beech trees. The nematodes recovered were identified under the microscope based on morphology and molecular analyses. Lcm nematodes from the funnels were counted under the microscope, and the number of nematodes was compared with the weather variables. The data was analyzed using R, and the generalized linear model based on negative binomial distribution was selected as the best fit model. Created with BioRender.com.

This study was conducted in the Stone Valley Forest, Pennsylvania (40°37’51.3” N and 77°53’08.03” W), under a research agreement with the Pennsylvania State University’s Forestlands Management Office. The site consists of a forest stand with BLD-symptomatic American beech trees (*F*. *grandifolia*). To understand the natural local dispersion of Lcm, samples were collected from September 9 to November 23, 2023. This period coincides with the migration of nematodes from highly infected leaves to newly forming buds [14]. To capture nematodes’ potential spread, four wooden funnel stands fixed in the ground were distributed as shown in Fig 1. Each funnel stand consisted of three funnels fixed horizontally at the top of the funnel stand at a height of 40 cm from the ground. All funnels were of uniform size, with an opening of 13 cm in diameter. To set an initial pilot test, two funnel stands were set up on September 9 at 3.51 m (stand 1) and 2.20 m (stand 2), respectively, at varying distances from the trunk of the closest BLD symptomatic beech trees. The diameter at breast height (DBH) of the nearest BLD symptomatic trees to funnel stands 1 and 2 was 50 cm, and 46 cm, respectively. Afterward, two additional sets of funnels were placed. On September 17, funnel stand 3 was set up at a distance of 3.51 m (at similar distance proximity as funnel stand 1) from the closest BLD symptomatic beech tree with a DBH of 5.6 cm. On September 28, the experiment was extended with funnel stand 4 located at 11.74 m distance from the closest infected beech tree with a 5 cm DBH. Each funnel was filled with tap water and covered with a wire mesh.

### Sample collection and nematode counts

Every other day, 50 ml of the water was collected in vials from each respective funnel (Fig 1). Once in the lab, the vials were kept vertically and straight for at least 1 hour, to allow enough time for the nematodes to settle down. The supernatant water was carefully discarded up to 10 ml, and nematodes were then transferred to a counting dish and counted under an inverted microscope (Axiovert A1, Carl Zeiss Microscopy, LLC, New York). Nematodes were initially identified morphologically in agreement with their trophic group, or to the genus level. Lcm observed moving were recorded as active, while those that did not move for 10 seconds were recorded as inactive. The total number of other nematodes was also recorded.

### Molecular identification of nematodes recovered from the funnels

Nematodes morphologically identified as belonging to the genus *Litylenchus* were hand-picked individually under the dissecting microscope (Stereo Discover V20, Carl Zeiss Microscopy, LLC, New York). Single nematode DNA extractions were performed using the Invitrogen PureLink Genomic DNA Mini kit (Invitrogen, Waltham, MA). Molecular identification was performed by amplification of the D2-D3 expansion segment of the large subunit (LSU) 28S rRNA gene using the primers: D2A (5’– ACAAGTACCGTGAGGGAAAGTTG–3’) and D3B (5’–TCGGAAGGAACCAGCTACTA– 3’) according to De Ley et al. [22] The PCR master mix consisted of 2.5 μl of 10X buffer (Invitrogen, Waltham, MA), 0.75 μl of 50 mM MgCl_2_ (Invitrogen, Waltham, MA), 2.5 μl of 10 mM dNTP Mix (Thermo Scientific, Waltham, MA), 0.1 μl of Platinum Tm Taq DNA polymerase (Invitrogen, Waltham, MA), 2 μl of template DNA, 0.75 μl of both forward and reverse primers, and water in 25 μl reaction. The PCR reactions were carried out in Bio-Rad T100 Thermal Cycler (Bio-Rad, Hercules, CA), with denaturation at 94°C for 2 minutes, followed by 94°C for 30 seconds, 55°C for 1 minute and 72°C for 2 minutes (39 cycles), and final extension at 72°C for 7 minutes. Molecular identification of nematodes other than *Litylenchus* sp. recovered from the funnels was performed using the universal primers targeting a fragment of the 18S rRNA gene: 18S F (5’-CGCGAATRGCTCATTACAACAGC-3’) and 18S R (5’-GGGCGGTATCTGATCGCC-3’), and the PCR reactions were performed following the methodology by Floyd et al. [23].

The PCR product lengths were confirmed in a 2% agarose gel followed by electrophoresis at 110 V for 40 minutes. The PCR products were purified with the QIAquick PCR purification kit (QIAGEN, Germantown, Maryland) and sequenced by Sanger Sequencing using the D2A and D3B primers for *Litylenchus* sp., and the 18SF and 18SR primers for other nematodes (Huck Institutes’ Genomics Core Facility at Penn State).

### Environmental variables data collection

Data for several environmental parameters were collected from the Stone Valley Forest to establish a potential correlation with the spread of Lcm at a local scale. The following variables were considered: humidity, average temperature, wind speed, and precipitation (rain). The data was recorded from the National Weather Service station (KAOO) at Huntingdon (PA), available at: https://www.weatherforyou.com. The values of each weather parameter taken from 8 Sept. through 8 Nov. are shown in S1 Table.

### Statistical analysis

The generalized linear models (GLMs) were used to determine if weather influences the dispersal of nematodes at the local scale. All three funnels from every funnel stand, and all counts of Lcm (including active and inactive), for every sampling date were pooled for these analyses. With the response variable being the total of Lcm, the predictors used in this model were the four funnel stands (at varying distances from the BLD symptomatic beech trees), average humidity, average wind speed, average precipitation, and average maximum temperature. To address the issue of overdispersion, the best-fitted model was obtained via negative binomial error distribution using ‘glm.nb’ function of the ‘MASS’ package. A backward selection procedure using likelihood ratio tests was used [24] to select the best combination of predictors. Model assumptions were assessed based on residual plots. Tukey HSD tests for multiple comparisons between treatments were employed with estimated marginal means comparisons (EMMs) using the “emmeans” function [25]. All statistical analyses were conducted in R (Version 2023.09.1+494).

### Insect caterpillars associated with BLD symptomatic leaves

Beyond venturing into the possible direct dissemination pathways of Lcm spread locally, the possibility of Lcm traversing unscathed the digestive systems of local fauna was explored. Six caterpillars identified on BLD symptomatic leaves during the established period (September to November) were collected to evaluate the association of nematodes with other interacting organisms. The collected caterpillars were brought to the laboratory for identification and kept overnight with BLD symptomatic leaves in a ziplocked plastic bag. The insect frass was collected the next day and kept in water for 30 minutes. Then, the frass was homogenized and soaked in water for another 3 hours. Afterward, the resulting mixed solution was observed under an inverted microscope for the presence of nematodes. The number of nematodes was counted and recorded as active or inactive. Single nematodes were processed for DNA extraction, and molecular identification was performed using the primers D2A and D3B [22]. The PCR reactions and corresponding sequencing methods were performed using the conditions described above.

### Spider webs collected under the canopies of BLD symptomatic trees

Two spider webs collected from the branches of BLD symptomatic trees were placed in 50 ml conical tubes and filled with 30 ml of water. After returning to the lab, the tubes were kept undisturbed and vertically for at least 1 hour, allowing nematodes to settle at the bottom of the vial. The collected nematodes were then observed and counted under an inverted microscope. Molecular nematode identification was carried out using the D2A and D3B primers [22].

## Results

### Pattern of nematodes recovered from funnels established apart BLD symptomatic trees

**Fig 2:**
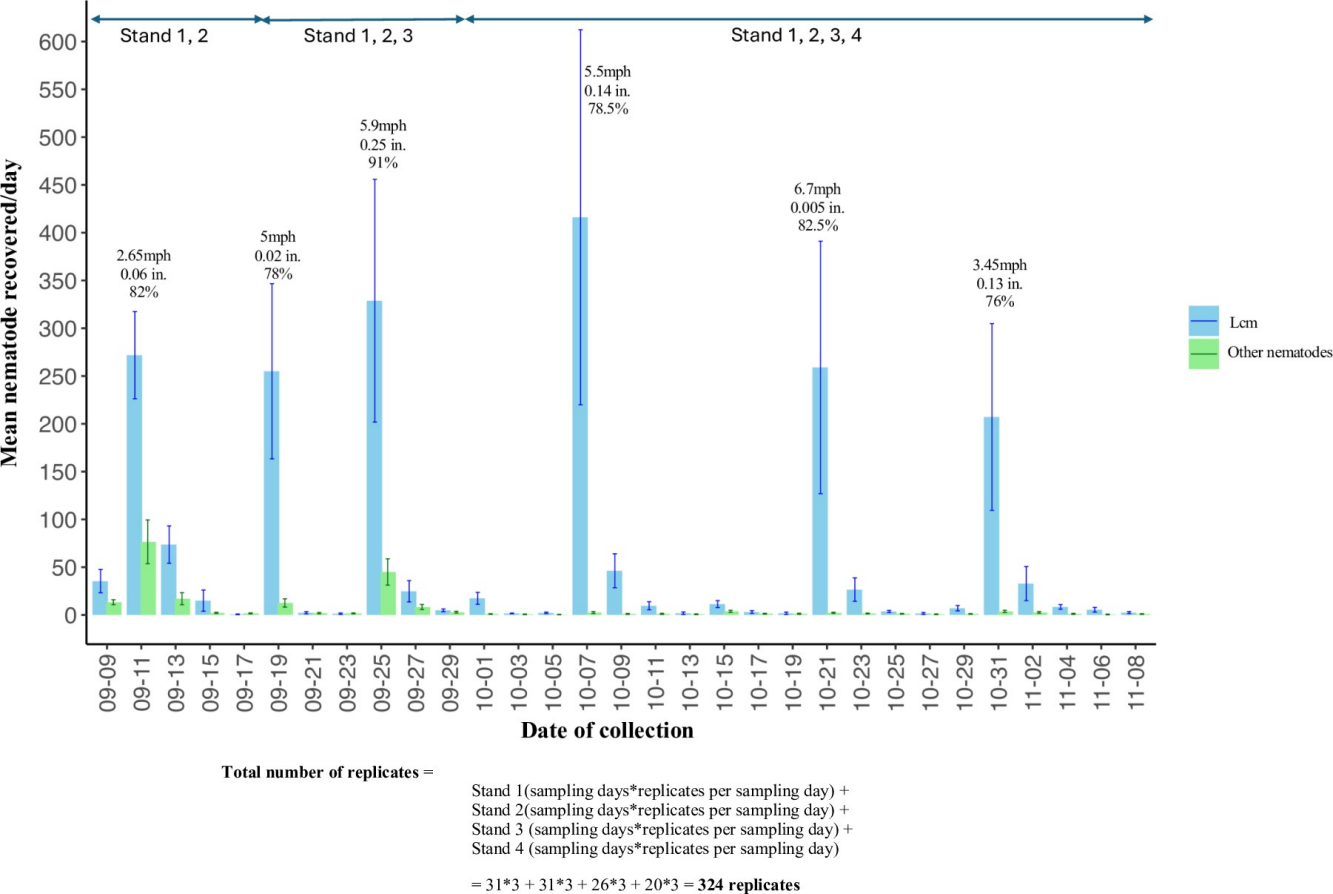
Total mean number of nematodes recovered on each sample collection day. The graph represents the total mean number of nematodes recovered from all the 12 funnels distributed in four stands. The blue color bars represent the mean numbers of *Litylenchus crenatae* spp. *mccannii* (Lcm), and the green bars represent nematodes other than Lcm recovered in a single day. Other nematodes include *Aphelenchoides* spp., *Laimaphelenchus* sp., *Diastolaimus* sp., *Panagrobelus* sp., *Mermithidae* sp., and *Geomonhystera* sp. The average values of the two days for parameters such as wind speed, rainfall, and relative humidity are presented in units of mph, inches, and percentage, respectively.

**Fig 3:**
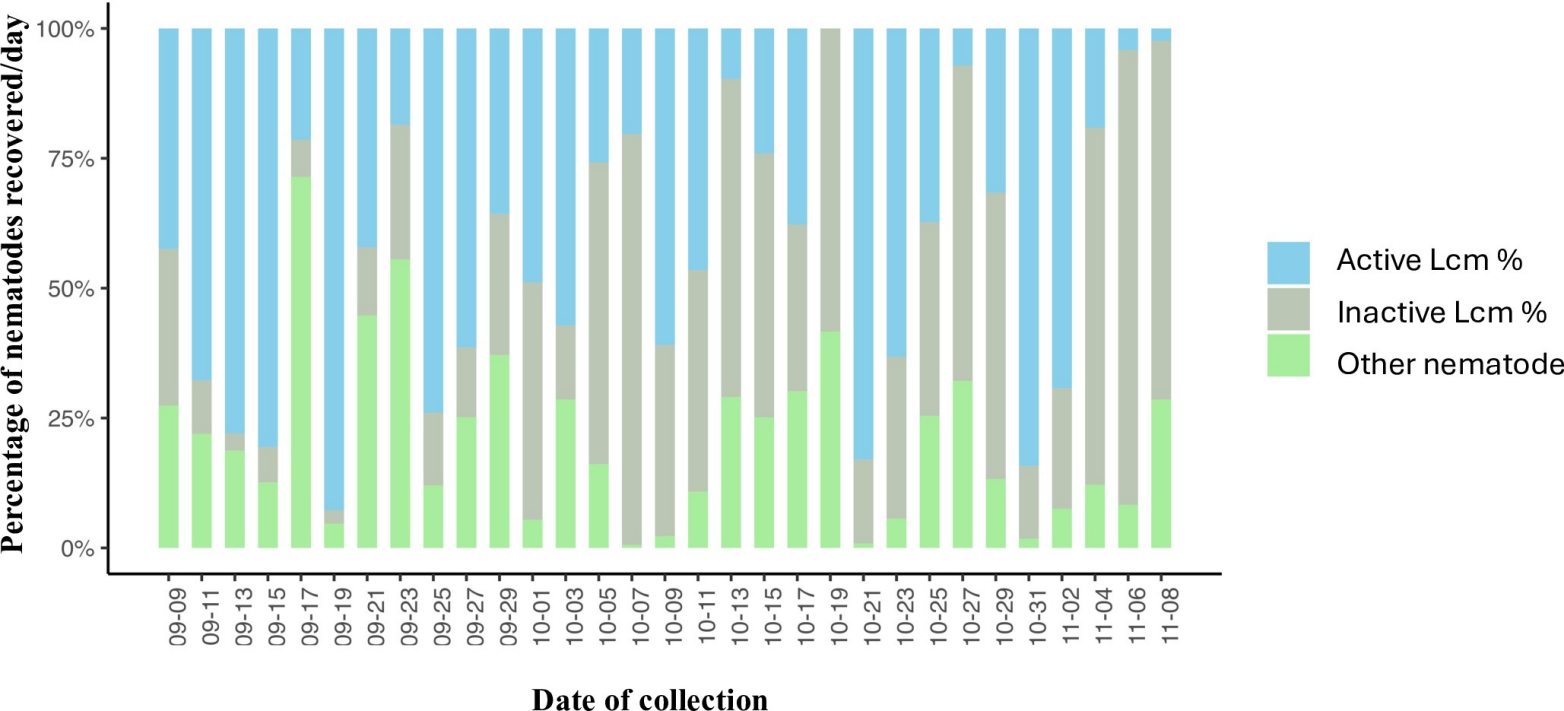
Percentage of active and inactive *Litylenchus crenatae* subsp. *mccannii*, and other nematodes, collected from all the funnels at each sampling day. Other nematodes include *Aphelenchoides* sp., *Laimaphelenchus* sp., *Diastolaimus* sp., *Panagrobelus* sp., *Mermithidae* sp., and *Geomonhystera* sp.

Wind can drive the spread of nematodes in nature [17, 26–28]. To assess the potential spread of Lcm by wind in a natural beech forest, funnels were placed at different distances from confirmed BLD symptomatic trees (Fig 1). From the 324 collected funnel samples, 266 (82%) samples revealed the presence of nematodes. The number and distribution of nematodes collected at each sampling date are shown in Fig 2. Our results revealed a high variation in the number of Lcm collected throughout the study, with notable peaks in nematode recovery from the funnels in September (11, 19, and 25), and October (7, 21, and 31). The number of recovered Lcm varied from a few individuals (as low as 1 nematode per single funnel sample) to several hundred individuals on most days. The highest number of Lcm recovered from a single funnel was 2,452 observed in October (Fig 2). Remarkably, up to 92% of the total nematodes recovered from the funnels throughout the study were identified as Lcm (Fig 3). Both active and inactive Lcm were counted. While a large percentage of Lcm were inactive, depending on the sample, up to 67% of Lcm recovered were active (Fig 3). A considerable percentage of active and inactive Lcm was observed across the sample collection dates except on October 19, when we did not recover any active Lcm (Fig 3). A smaller proportion of other nematodes were also captured within the funnels but on a much smaller scale (about 8% of the total of nematodes). These nematodes were distributed across different genera, mostly classified as free-living nematodes (S2 Table).

To validate the morphological identification, a molecular diagnosis was performed for four *Litylenchus* specimens, one individual per funnel stand, using a region of the 28S rRNA gene. The amplification yielded fragments of 741 bp flanked by the D2A and D3B primers (GenBank accession no. PP989820). The sequences generated for all specimens revealed 100% similarity to *L*. *crenatae* ssp. *mccannii* sequences obtained from specimens collected from other geographical areas in the US (accession numbers: MK292138, MK292137, MT0791193, MN525396, MN525397), and 95.35% similarity to *L*. *coprosma* (KY679564.1), therefore, validating our morphological analyses. The remaining nematodes were molecularly identified based on the 18S rRNA region and distributed along the following taxa, *Aphelenchoides* sp., *Laimaphelenchus* sp., *Diastolaimus* sp., *Panagrobelus* sp., *Mermithidae* sp., and *Geomonhystera* sp. (S2 Table).

### Distance and DBH impact on the Lcm dispersion

**Fig 4:**
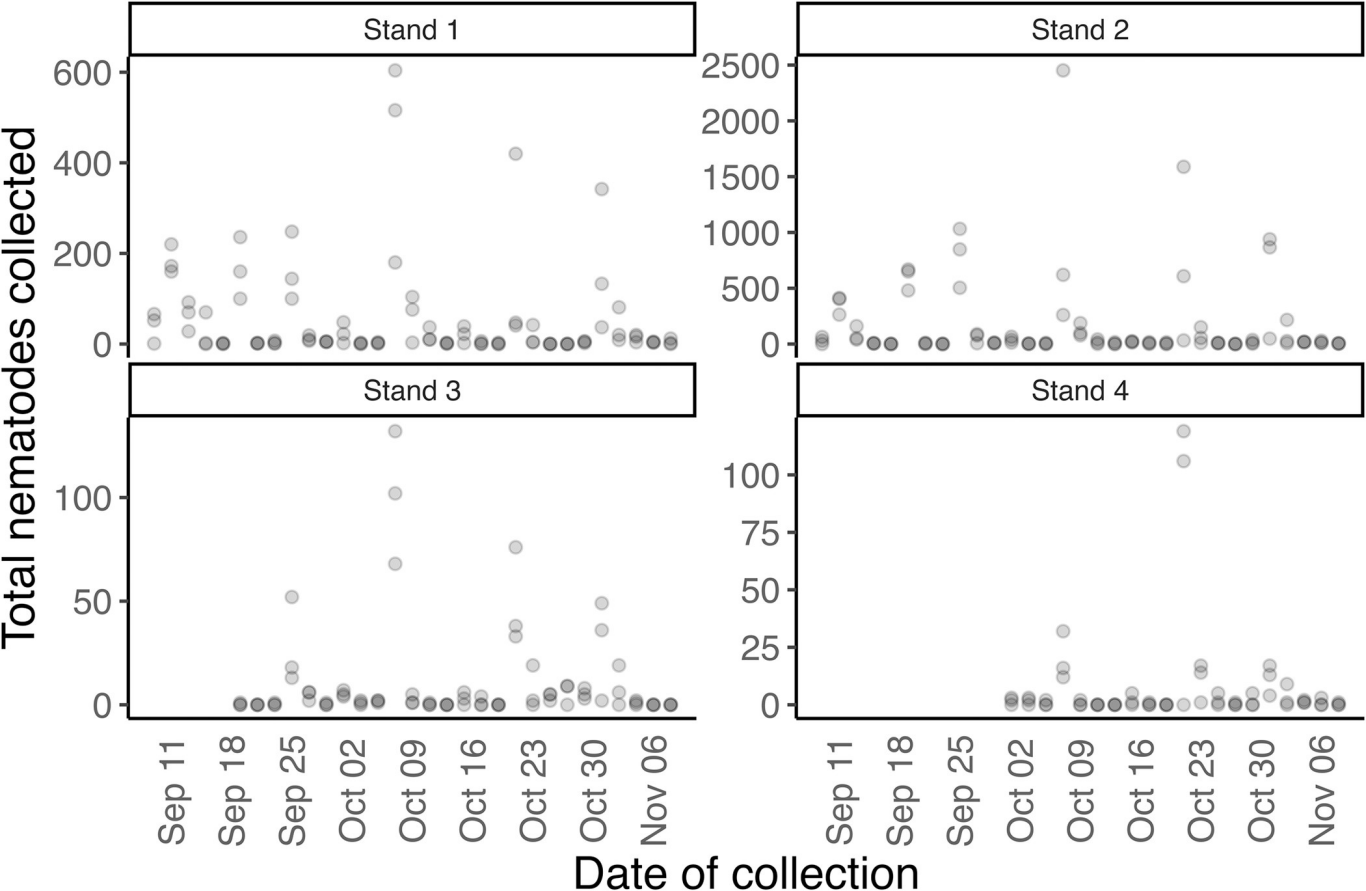
Nematode counts for all the funnels in the four funnel stands. Every single dot represents *Litylenchus crenatae* subsp. *mccannii* counts from each funnel counted on every second day from September 9 to November 8. The light gray dots represent nematode numbers counted from single funnels. Dark gray represents two or more funnels with the same number of nematodes recovered.

**Fig 5:**
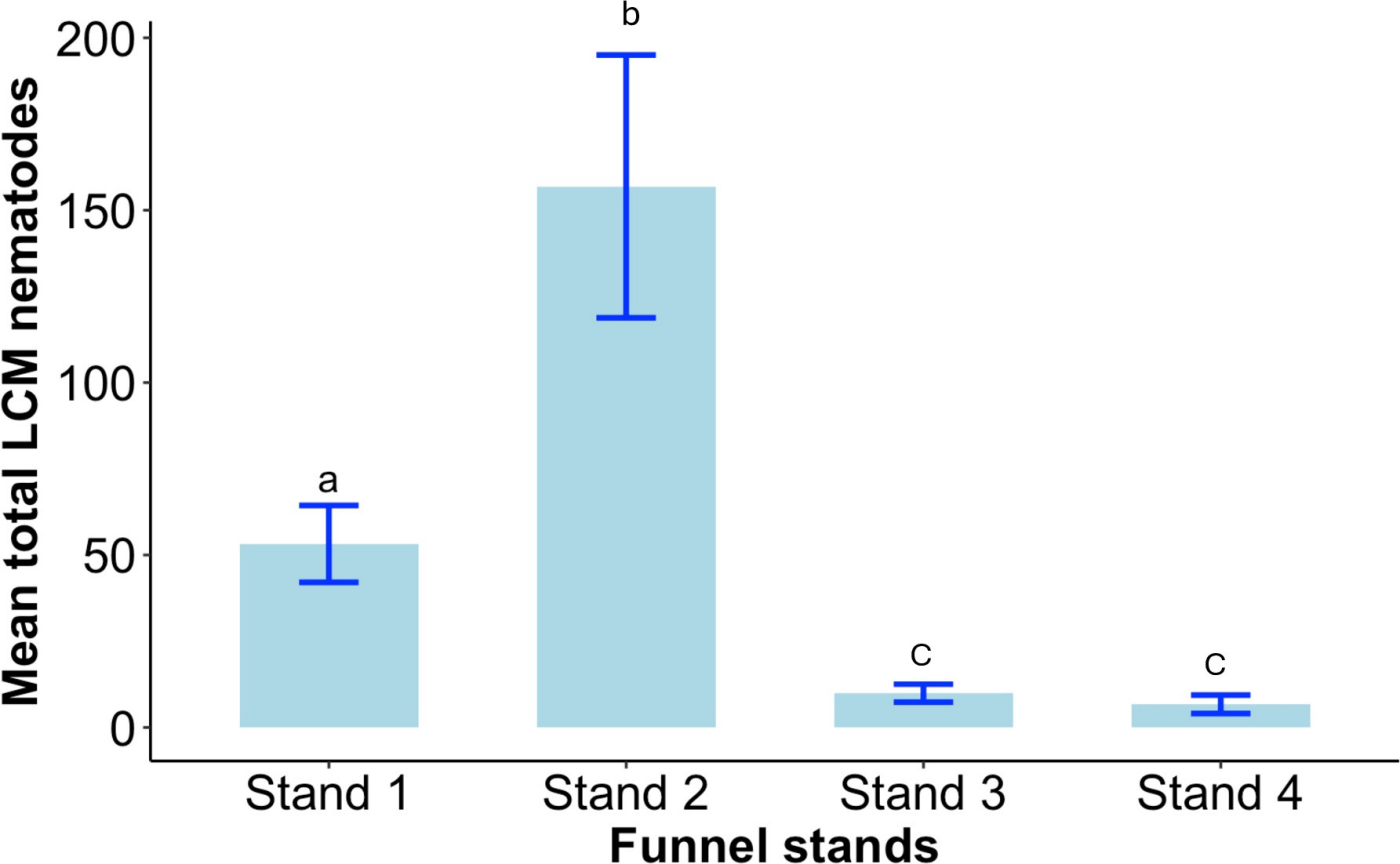
Comparison of the mean *Litylenchus crenatae* subsp. *mccannii* recovered from the funnel stands at different proximity of the BLD infected beech trees with variable radial growth measured as diameter at breast height. Bars with the same letter are not significantly different (p > 0.05).

Based on the samples collected from the funnels, we observed variations in the number of nematodes within each funnel stand suggesting a potential correlation with the distance and DBH of the closest symptomatic tree (Fig 4). The BLD-infected beech tree with a DBH of 46 cm closest to funnel stand 2 (i.e., 2.2 m), exhibited the highest number of Lcm nematodes throughout the experiment, ranging from 0 to 2,452 specimens. Funnel stand 1 was located 3.51 m from the nearest BLD-infected tree with a DBH of 50 cm, and Lcm nematodes recovered from these funnels ranged between 0 to 600. This indicates the Lcms can easily be dispersed to the nearby trees within the range of several meters apart from a symptomatic tree (Fig 4). This was further supported by the approximately same level of BLD infection between trees 1 and 2. More specifically, significant differences were observed in the Lcms recovered from the funnels of funnel stands 1 and 2 (Fig 5). The nearest trees to stands 1 and 2 presented medium-high BLD symptoms, with over half of the leaves presenting dark-green interveinal bands.

In addition to distance, the effect of DBH was also observed to impact the number of Lcm nematodes recovered from the funnels. Another funnel stand, referred to as funnel stand 3, was set up on September 17, at a similar distance from the closest infected tree (3.51 m) as funnel stand 1, but with a much lower DBH of 5.6 cm. The number of Lcm nematodes recovered from this stand ranged from 0 to 132 (Fig 4). These results were further supported by significant differences between funnel stand 1 and funnel stand 2 compared to funnel stand 3 (Fig 5).

Furthermore, we investigated the nematode recovery in the funnels at a further distance from closely infected trees with lower DBH. Funnel stand 4 was set up on September 30, at 11.74 m from the nearest BLD-infected beech tree with a DBH of 5 cm. Interestingly, we recovered almost the same number of Lcms (between 0 to 119) nematodes, despite having over 3 times the distance compared to funnel stand 3. The nearest trees to stands 3 and 4 presented low BLD symptoms, with only a few leaves presenting dark-green interveinal bands.

The data indicate that nematodes could be dispersed at least 11.74 m from the nearest BLD-infected tree (Fig 4). Funnel stand 4 did not show any significant difference from stand 3 in the total Lcm recovered, but it showed significant differences from funnel stands 1 and 2 (Fig 5). These results highlight the influence of both the distance from the infected tree and the DBH of the tree on the dispersal of Lcm.

### Environmental variables can directly impact local Lcm dispersal

Several abiotic variables, such as precipitation, humidity, wind speed, and maximum temperature, were considered to explore their potential impact on the local dispersion of Lcm (S1 Table). Table 1 presents the best-fit model summary derived from GLM analyses, selected based on the lowest Akaike information criterion (AICc) value, assessing the relationship between the total number of Lcm and various abiotic variables.

**Table 1:** A generalized linear model using the negative binomial distribution.

Variables	Estimate	Standard error	z value	p value
(Intercept)	-4.00	1.45	-2.76	0.0058
Funnel Stand 2	0.93	0.25	3.64	0.0002
Funnel Stand 3	-1.63	0.27	-5.92	0.0000
Funnel Stand 4	-2.04	0.30	-6.73	0.0000
Temperature maximum	0.06	0.01	0.46	0. 6476
Humidity	0.06	0.01	4.91	0.0000
Wind speed	0.46	0.06	7.32	0.0000
Precipitation	21.29	3.34	6.37	0.0000
Wind speed: Precipitation	-3.23	0.54	-5.94	0.0000

The response variables were the total number of *Litylenchus crenatae* subsp. *mccannii* (active as well as inactive) in the four funnel stands and the possible predictors were the date of collection, funnel stands 1 to 4, humidity, average temperature, wind speed, and precipitation. The presented model is the best-fitted model for the data.

**Fig 6:**
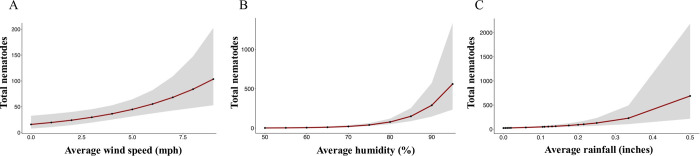
Prediction graphs for *Litylenchus crenatae* subsp. *mccannii* (Lcm) dispersal influenced by wind speed, humidity, and precipitation. **A.** Prediction of the number of Lcm impacted by the wind speed. **B**. Prediction of Lcm nematode recovery impacted by the average humidity. **C.** Prediction of the Lcm number influenced by precipitation in the form of rain.

The model utilized a logarithmic link function for the estimated contrasts. The interpretation of the estimated contrasts, z values, and the p values of the best-fit model revealed that the relative humidity and wind speed have a positive effect on the number of Lcm recovered from funnels (Table 1). It also predicted a significantly higher output of Lcm numbers with higher precipitation, whereas the predictor estimate was less reliable because of the standard error (Table 1). A negative effect of wind speed and precipitation interaction on Lcm number was predicted by the model. The model did not show any significant influence of maximum temperature on the nematode number at this time of the year (Table 1). Therefore, according to the best-fit model with the increase in wind speed, humidity, and precipitation (rainfall), the number of Lcm also increases (Fig 6).

When an analysis of variance (ANOVA) was conducted to determine the significance of the predicted effects of variables by the best-fitted model on Lcm dispersal, we found a significant impact of funnel stand, humidity, wind speed, and interaction of wind speed and precipitation on the number of Lcm (Table 2). Significant differences were observed when all funnel stands were compared for the total of Lcm collected. We also observed a significant positive relationship between the number of Lcm collected from the funnels with humidity and wind speed (Table 2). A significant negative interaction between wind speed and precipitation was observed in the number of Lcm collected (Table 2), indicating the effect of wind speed becomes less positive as precipitation increases or vice versa. Furthermore, there was no significant influence of maximum temperature on the quantity of nematode recovered in the funnels between Sept. 9 and Nov. 8 (Table 2). Although increased precipitation led to a higher output of Lcm numbers (Fig 6C), the effect was non-significant (Table 2).

**Table 2:** ANOVA of the best fit generalized linear model using the negative binomial model.

Variables	Degrees of freedom	Chi square value	p value
Funnel stand	3	115.67	**0.0000**
Temperature maximum	1	0.19	0.6615
Humidity	1	20.90	**0.0000**
Wind speed	1	18.72	**0.0000**
Precipitation	1	3.19	0.0747
Wind speed: Precipitation	1	12.16	**0.0048**

p values < 0.05 are shown in bold.

### Unspecific association of Lcm with other organisms

Following the results above, we explored the potential link between Lcm and other organisms commonly associated with the American beech ecosystem considering the possibility of nematode spread through wind-mediated mechanisms. Therefore, additional samples were collected from organisms interacting with the tree canopy, such as caterpillars (B), and from suspended aerial structures like spider webs (Fig 1C).

A total of six different caterpillars associated with BLD symptomatic trees were collected and processed in the lab. Among the six caterpillars examined, the frass of the Tussock moth caterpillar (*Halysidota tessellaris*) was observed to contain 12 nematode specimens. Among these, 10 were identified as Lcm (2 actively moving and 8 in inactive state), while the remaining two were identified as free-living nematodes. The frass collected from other caterpillars (*Amphipyra pyramidoides*, *Anisota senatoria*, *Phlogophora* sp., *Campaea perlata*, and *Symmerista canicosta*) was absent of nematodes. Nematode molecular identification using the D2D3 region of the 28S rRNA gene of one individual confirmed the identification of Lcm (accession number: PQ002232). The presence of active Lcm in the caterpillar frass indicates that Lcm can survive the passage through the caterpillar’s gastrointestinal tract, thus, suggesting that caterpillar feeding activity from BLD symptomatic leaves can potentially contribute to local dispersal of Lcm.

To further verify the dispersal and incidental association of Lcm with other organisms, natural “pending structures” like spider webs were collected. Lcm were recovered from two spider webs collected from the branches of a BLD-infected beech tree. A total of 255 individuals were recovered from a single spider web, with 58 actively moving and 197 in an inactive state. From the other spider web, there were 10 actively moving Lcm nematodes and 24 in inactive state. Using molecular identification based on the D2D3 region, these nematodes were confirmed as Lcm (accession number: PP991509), validating their presence on the spider webs.

## Discussion

As BLD continues to spread rapidly across the Northeastern United States and Ontario province of Canada [12, 13], it is crucial to understand the dispersal mechanism(s) of its causal agent, Lcm. This study is the first to use the Baermann funnel method in a forest setting to capture live PPNs without an extraction method. Although various hypotheses have been proposed regarding the possible means of Lcm dispersal, including windborne rain, mites, beetles, and birds [29, 30], there is currently insufficient empirical data to support or refute these hypotheses. To the best of our knowledge, this is the first attempt to quantify the influence of a collective number of variables on the local spatial distribution of Lcm. The current study explores various dispersal mechanisms, including distance to BLD symptomatic trees, tree size, and weather variables. As a proof of concept, we also investigated the potential interaction of Lcm with other biological agents as a preliminary assessment of their dispersion capabilities.

The influence of wind on nematode dispersal has been reported across various natural ecosystems. Free-living nematodes constitute the most prevalent taxa in natural habitats (i.e., non-agriculture systems) [31]. Our data, however, consistently showed higher recovery of Lcm, a forest PPN, during the fall season, compared to the sporadic and lower occurrence of other nematodes throughout the study. A small percentage of foliar (e.g., *Aphelenchoides* sp.) and free-living nematodes were also recovered from the funnels, probably reflecting the natural diversity of nematodes in our study area. The recovery of free-living nematodes aligns with a study conducted in Central Europe, which identified free-living nematodes as the predominant organisms recovered through wind-mediated dispersal [28]. Similarly, a month-long investigation in Antarctica identified nematodes as the main taxa among wind-dispersed invertebrates [32]. In contrast to the findings of previous studies, Lcm accounted for the majority of nematodes recovered from the funnels. These results are particularly noteworthy due to their unexpected nature, especially considering they are exclusively plant-parasitic nematodes. Furthermore, the high percentage of active Lcm found within the funnels suggests that this nematode will likely survive and thrive in these environmental conditions. The preponderance of Lcm indicates a robust active movement, reflecting the migration of nematodes from leaves into the buds. It also highlights their high population density (above 90% of the nematodes recovered were Lcm) at this time of the year. Based on the substantial number of Lcm recovered in our study, it is evident that wind plays a significant role in the local dispersion of this nematode.

The dispersal of Lcm was also investigated in response to the proximity to BLD symptomatic trees. Funnel stands 1 and 2 were positioned at close distance from highly infected trees (3.51 m and 2.20 m, respectively), and showed significant differences in the counts of Lcm. The number of nematodes recovered from stands 1 and 2 was notably higher compared to stand 4, located further away (11.74 m) from the nearest symptomatic beech tree. These findings suggest a strong correlation between the proximity to the disease source (the tree) and the dispersal of nematodes, as higher nematode numbers were recovered closer to the infected tree. However, even at a greater distance from the tree, Lcm nematodes were still found.

Besides distance, another important factor that influenced the number of nematodes was the width (DBH) of the tree, i.e., larger trees possess larger canopies, larger number of leaves, and ultimately longer branches. Funnel stands 1 and 3 were placed equidistantly at 3.51 m from their respective nearby infected trees. The major distinction lay in the DBH diameter of 46 cm compared to 5.6 cm, respectively. When comparing the nematode counts from these two stands we observed significant differences in the number of nematodes recovered from stand 1 compared to stand 3. The results suggest that trees with larger canopies can host more nematodes due to their increased foliage, which enhances their potential for nematode inoculum, and have a stronger participation in nematode dispersal to the surrounding areas. Lastly, there were no significant differences between stands 3 and 4, located at variable distances of 3.51 m and 11.74 m, respectively, from their closely infected beech trees. Although both trees possess a similar DBH (5.6 cm versus 5 cm), nematode presence was comparable in both cases. Overall, the size directly impacts the number of recovered nematodes, with the wind playing a pivotal role in their local distribution. This suggests a complex dispersal pattern wherein proximity to the source amplifies nematode presence but does not preclude their occurrence at more distant locations from smaller symptomatic trees. Understanding these dynamics, particularly the extent to which wind can disseminate this nematode over long distances, are of critical importance.

In addition to wind speed, we also evaluated the effect of temperature, humidity, and precipitation, to assess their impact on Lcm dispersal. We employed multiple predictive models to estimate nematode dispersal by various weather conditions. The best-fit model suggests that wind speed and humidity have high positive significance in mediating nematode dispersal, indicating that increases in these variables correlate with higher Lcm counts in the environment. Similarly, other studies observed a notable influence of weather factors such as wind speed, relative humidity, and raindrops on the dispersal of different plant pathogens in the environment [33–35].

While wind speed and humidity are important factors determining the spread of Lcm, the current study’s results also showed a slight positive influence of precipitation on nematode dispersal. However, this impact is somewhat mitigated, likely due to a pronounced negative interaction between wind and rain. At low precipitation levels, increased wind speed might facilitate Lcm dispersal. However, as precipitation increases, the positive effect of wind speed diminishes or potentially becomes negative. The trend of negative interaction between wind and rain was particularly noticeable on October15, when wind speeds reached 7 mph with gusts up to 21 mph, coupled with 0.5 inches of rainfall. This was likely due to higher wind speeds, which could have dispersed the Lcm nematodes over greater distances. As a result, fewer nematode numbers were found in the funnels closer to the BLD-infected trees. In addition, heavy rain could impact on the wide dispersion of Lcm, it may also exert a significant preponderance of dispersing the nematode from high elevations to the ground. This dual effect highlights the complex interplay between weather patterns and the population dynamics of nematode dispersal.

We did not observe any significant influence of temperature on nematode dispersal at this time of the year. Optimal wind speeds could have the potential to greatly enhance the spread of Lcm over longer distances. Hence, wind speed can be used as a strong indicator of Lcm dispersal into new potential beech forest areas. Overall, the study of weather variables impacting pathogen dispersal models enhances our comprehension of the broader implications of weather conditions on disease dispersal and monitoring efforts [36].

Lastly, we hypothesized that any biotic form with the ability to move from a BLD-infected tree would be able to disperse the Lcm nematode to other non-infected trees. This hypothesis was based on the premise that Lcm can migrate along the leaf surfaces and twigs, particularly during periods of high population density at this time of the year [14]. Thus, Lcm could potentially “hitchhike” on various organisms. As proof of concept, we evaluated the presence of Lcm on organisms, or their derivate structure, commonly found in the forest. To test this hypothesis, we first collected different caterpillars from the leaves of BLD symptomatic trees. Remarkably, we successfully recovered live Lcm specimens from *H*. *tessellaris* caterpillar frass. This indicated the ability of Lcm to pass through the caterpillar gut undigested, implying that caterpillars may indirectly aid in the dispersal of Lcm. It is also tempting to speculate that birds while consuming these caterpillars as a source of nutrients, might also contribute to the dispersal of the nematode. Following this scenario, a much larger percentage of bird species could potentially contribute to the long-distance dispersal of the nematode by feeding on caterpillars carrying the nematodes. This complements the current hypothesis that a more restricted number of bird species (e.g., finches) utilize beech buds as food resources [30]. Further research is necessary to validate the survival of the nematode through the avian digestive system.

Then, we turned our attention to spider webs in the vicinity of BLD trees. Interestingly, a high number of active Lcm nematodes were recovered from these randomly collected webs. The unspecific association of BLD with these natural “pending structures” can be interpreted in two different ways. First, nematodes may fall under gravity’s influence, potentially spreading the infection to other trees beneath the canopy. In fact, extensive observations suggest that younger trees are often heavily infected with BLD and are frequently the first to exhibit BLD symptoms. Second, if nematodes are found in spider webs, there is a strong likelihood that they may be transported by other “incidental organisms” (e.g., insects, birds, mammals), thereby potentially increasing the number and impact of nonspecific nematode vectors.

## Conclusions

This study uncovers critical abiotic conditions that could play an important role in the local dispersal of Lcm. Overall, our data indicates that the complexity of different factors, such as the distance and size of infected beech trees, wind speed, and humidity significantly impact the dispersal of nematodes. Moreover, the multitude of organisms interacting beneath the canopy can also contribute to the local dispersion of Lcm. Although the current study sheds light on some of the gaps in BLD local dispersion, some major questions still need to be addressed in future studies. One critical area is understanding how Lcm nematodes navigate factors such as wind, humidity, and precipitation to facilitate the spread of BLDs. Another important question to be addressed is the survival capacity of Lcm (e.g., anhydrobioses state), when transmitted in long-distance ranges, whether transmitted by abiotic or biotic vectors. Our results suggest that tree size can significantly impact the abundance of nematodes in the environment, promoting their dispersal to lower beech trees/seedlings. In conclusion, our study has demonstrated the influence of various factors shaping the local dispersal dynamics of Lcm. The insights gained by this study not only enhance our understanding of Lcm local scale dispersal patterns but also lay a foundation for predicting its potential long-range dispersion.

## Supporting information

S1 Table Abiotic variables investigated to study their potential impact on the Lcm dispersal. Temperature (maximum, minimum, and average), wind speed and wind gusts, humidity, and precipitation is presented in units of F, mph, inches, and percentage, respectively.
(DOCX)

S2 Table Other nematodes than Lcm detected in this study recovered from the funnels. (DOCX)
