# Supplementary material for: Deciphering the vectors: Unveiling the local dispersal of Litylenchus crenatae ssp. mccannii in the American beech (Fagus grandifolia) forest ecosystem
Source: PLoS One. 2024 Nov 8;19(11):e0311830. doi: 10.1371/journal.pone.0311830 (PMC11548727; doi:10.1371/journal.pone.0311830)
Supplement: S1 Table — Temperature (maximum, minimum, and average), wind speed and wind gusts, humidity, and precipitation is presented in units of F, mph, inches, and percentage, respectively. (DOCX) [file pone.0311830.s001.docx]

**Supplementary material**

**S1 Table.** Abiotic variables investigated to study their potential impact on the Lcm dispersal

| **Date** | **Temp max** | **Temp**  **min** | **Avg**  **temp** | **Wind**  **speed** | **Wind**  **gusts** | **Humidity** | **Precipitation** | |
| --- | --- | --- | --- | --- | --- | --- | --- | --- |
| 9/8/23 | 79 | 64 | 71 | 2.7 | - | 81.00 | 0.10 |  |
| 9/9/23 | 82 | 63 | 69.3 | 2.8 | - | 85.00 | 0.33 |  |
| 9/10/23 | 76 | 63 | 69.4 | 1.9 | 25 | 83.00 | 0.09 |  |
| 9/11/23 | 79 | 57 | 68.2 | 3.4 | - | 81.00 | 0.03 |  |
| 9/12/23 | 80 | 53 | 65.8 | 3.2 | - | 78.00 | 0.23 |  |
| 9/13/23 | 74 | 58 | 65 | 5.3 | 24 | 72.00 | 0.01 |  |
| 9/14/23 | 70 | 48 | 58.2 | 4.4 | - | 73.00 | 0.01 |  |
| 9/15/23 | 71 | 43 | 56.5 | 3.8 | - | 71.00 | 0.01 |  |
| 9/16/23 | 76 | 42 | 57.2 | 2.9 | - | 73.00 | 0.00 |  |
| 9/17/23 | 60 | 47 | 55.6 | 1.1 | - | 89.00 | 0.22 |  |
| 9/18/23 | 70 | 53 | 58.8 | 3.6 | - | 83.00 | 0.03 |  |
| 9/19/23 | 71 | 49 | 57.8 | 6.4 | - | 73.00 | 0.01 |  |
| 9/20/23 | 74 | 46 | 58.4 | 2.6 | 23 | 73.00 | 0.01 |  |
| 9/21/23 | 74 | 45 | 52.4 | 1.3 | - | 83.00 | 0.00 |  |
| 9/22/23 | 69 | 42 | 52.1 | 3 | - | 79.00 | 0.00 |  |
| 9/23/23 | 55 | 52 | 54 | 8.7 | - | 87.00 | 0.40 |  |
| 9/24/23 | 63 | 54 | 58.1 | 7.6 | 21 | 92.00 | 0.41 |  |
| 9/25/23 | 65 | 56 | 59.6 | 4.1 | 17 | 90.00 | 0.09 |  |
| 9/26/23 | 56 | 53 | 54.8 | 5.3 | 18 | 91.00 | 0.20 |  |
| 9/27/23 | 67 | 48 | 56.3 | 4.9 | - | 70.00 | 0.01 |  |
| 9/28/23 | 67 | 43 | 54.5 | 3.7 | 17 | 77.00 | 0.00 |  |
| 9/29/23 | 64 | 57 | 59.9 | 1.3 | - | 85.00 | 0.00 |  |
| 9/30/23 | 74 | 55 | 63.8 | 2.3 | - | 78.00 | 0.00 |  |
| 10/1/23 | 78 | 50 | 57.3 | 1.3 | 17 | 85.00 | 0.00 |  |
| 10/2/23 | 78 | 50 | 57.2 | 0.6 | - | 86.00 | 0.01 |  |
| 10/3/23 | 81 | 51 | 59.7 | 1.3 | - | 84.00 | 0.01 |  |
| 10/4/23 | 80 | 52 | 60.1 | 1.2 | - | 84.00 | 0.00 |  |
| 10/5/23 | 75 | 49 | 60.4 | 3.7 | - | 78.00 | 0.00 |  |
| 10/6/23 | 70 | 61 | 63.4 | 2.4 | 17 | 84.00 | 0.22 |  |
| 10/7/23 | 60 | 47 | 54.9 | 8.6 | - | 73.00 | 0.06 |  |
| 10/8/23 | 54 | 42 | 48.2 | 11.1 | 25 | 61.00 | 0.00 |  |
| 10/9/23 | 59 | 39 | 48.2 | 6.7 | 29 | 67.00 | 0.00 |  |
| 10/10/23 | 63 | 42 | 52.2 | 5.9 | 18 | 63.00 | 0.00 |  |
| 10/11/23 | 67 | 40 | 52.5 | 4.9 | 20 | 58.00 | 0.00 |  |
| 10/12/23 | 69 | 39 | 54.8 | 2.2 | - | 67.00 | 0.01 |  |
| 10/13/23 | 64 | 42 | 51.1 | 2 | - | 69.00 | 0.00 |  |
| 10/14/23 | 49 | 45 | 46.8 | 5.2 | - | 91.00 | 0.89 |  |
| 10/15/23 | 52 | 44 | 46.9 | 8.7 | 18 | 80.00 | 0.11 |  |
| 10/16/23 | 55 | 42 | 48.4 | 6.3 | 24 | 78.00 | 0.02 |  |
| 10/17/23 | 52 | 48 | 49.7 | 4.6 | 16 | 76.00 | 0.02 |  |
| 10/18/23 | 54 | 39 | 48.8 | 0.8 | - | 74.00 | 0.00 |  |
| 10/19/23 | 63 | 35 | 43.9 | 1.7 | - | 83.00 | 0.01 |  |
| 10/20/23 | 56 | 51 | 54 | 4.8 | - | 87.00 | 0.28 |  |
| 10/21/23 | 54 | 47 | 50.8 | 8.6 | 18 | 78.00 | 0.07 |  |
| 10/22/23 | 55 | 42 | 46.9 | 11.5 | 28 | 65.00 | 0.02 |  |
| 10/23/23 | 61 | 35 | 45.2 | 4.7 | - | 63.00 | 0.01 |  |
| 10/24/23 | 73 | 35 | 51.5 | 2.6 | 22 | 62.00 | 0.00 |  |
| 10/25/23 | 80 | 42 | 58.6 | 2.9 | - | 54.00 | 0.00 |  |
| 10/26/23 | 78 | 44 | 61.4 | 3.6 | - | 53.00 | 0.00 |  |
| 10/27/23 | 75 | 51 | 63.7 | 4.1 | - | 67.00 | 0.02 |  |
| 10/28/23 | 72 | 60 | 65.8 | 6.7 | 18 | 76.00 | 0.04 |  |
| 10/29/23 | 60 | 52 | 53.5 | 2 | 21 | 90.00 | 0.63 |  |
| 10/30/23 | 57 | 41 | 51.1 | 4.6 | - | 84.00 | 0.26 |  |
| 10/31/23 | 45 | 31 | 38.7 | 2.3 | 26 | 68.00 | 0.00 |  |
| 11/1/23 | 40 | 30 | 35.1 | 9.3 | 17 | 72.00 | 0.03 |  |
| 11/2/23 | 47 | 26 | 35.6 | 4.2 | 28 | 61.00 | 0.00 |  |
| 11/3/23 | 61 | 26 | 42.3 | 4.4 | - | 52.00 | 0.00 |  |
| 11/4/23 | 61 | 34 | 47.7 | 3 | 17 | 50.00 | 0.00 |  |
| 11/5/23 | 63 | 41 | 53.2 | 6 | 16 | 50.00 | 0.00 |  |
| 11/6/23 | 60 | 37 | 49.5 | 5.6 | 22 | 61.00 | 0.00 |  |
| 11/7/23 | 69 | 45 | 60.3 | 12.5 | 22 | 50.00 | 0.01 |  |
| 11/8/23 | 56 | 36 | 45.8 | 4.2 | 33 | 82.00 | 0.00 |  |

Temperature (maximum, minimum, and average), wind speed and wind gusts, humidity, and precipitation is presented in units of F, mph, inches, and percentage, respectively.
