# Supplementary material for: Deciphering the vectors: Unveiling the local dispersal of Litylenchus crenatae ssp. mccannii in the American beech (Fagus grandifolia) forest ecosystem
Source: PLoS One. 2024 Nov 8;19(11):e0311830. doi: 10.1371/journal.pone.0311830 (PMC11548727; doi:10.1371/journal.pone.0311830)
Supplement: S2 Table — (DOCX) [file pone.0311830.s002.docx]

**Supplementary material**

**S2 Table.** Other nematodes than Lcm detected in this study recovered from the funnels

| **Other nematodes** | | **Sequence size** | **Matches with** | **Percent identity** | **q-value** |
| --- | --- | --- | --- | --- | --- |
| *Laimaphelenchus* sp. | 822 | | EU306346.1 | 98.41 | 100 |
| *Diastolaimus* sp. | 842 | | OM691517 | 94.19 | 100 |
| *Mermithidae* sp. | 808 | | MG182364 | 99.88 | 100 |
| *Geomonhystera* sp. | 817 | | LC382087 | 96.83 | 100 |
| *Panagrobelus stammeri* | 755 | | FJ969134 | 99.74 | 100 |
| *Aphelenchoides* sp. | 569 | | MT410633 | 87.93 | 81 |
